# Distinct trajectories of low anterior resection syndrome following ileostomy reversal

**DOI:** 10.3389/fmed.2026.1735910

**Published:** 2026-06-30

**Authors:** Xuena Zhang, Qingyu Meng, Jingru Wang, Simeng Jiang, Zhongtao Tian, Zihan Fan, Tong Wang, Wenbo Niu

**Affiliations:** 1 Department of Nursing, The Fourth Hospital of Hebei Medical University, Shijiazhuang, Hebei, China; 2 Department of General Surgery, The Fourth Hospital of Hebei Medical University, Shijiazhuang, Hebei, China

**Keywords:** bowel dysfunction, group-based trajectory modeling, low anterior resection syndrome, preventive stoma, rectal cancer

## Abstract

**Purpose:**

This study is an midterm postoperative assessment of LARS symptoms, aiming to apply group-based trajectory modeling to analyze the symptom trajectories of low anterior resection syndrome in rectal cancer patients who underwent ileostomy and subsequent reversal, and to investigate the clinical and treatment-related factors influencing these trajectories.

**Methods:**

A retrospective cohort of 134 rectal cancer patients who underwent low anterior resection with diverting ileostomy and subsequent reversal was analyzed. LARS scores were recorded at 3, 6, 9, and 12 months postoperatively. GBTM was used to classify patients into symptom trajectory groups. Multinomial logistic regression identified clinical factors associated with each trajectory.

**Results:**

Among 134 rectal cancer patients included, group-based trajectory modeling identified three LARS symptom trajectories following stoma reversal: mild and recovering (30.6%), moderate and fluctuating (40.3%), and severe and persistent (29.1%). Over the 12-months follow-up, LARS scores declined overall, with significant differences in symptom severity observed among the three groups at all time points (*P* < 0.001). Most patients in the mild trajectory group improved steadily and had no LARS by 12 months, whereas nearly half of the patients in the severe trajectory group continued to experience major LARS. Multivariable analysis revealed that higher T stage, lower anastomotic height, and receipt of preoperative radiotherapy were independently associated with worse trajectories.

**Conclusion:**

Preoperative radiotherapy, low anastomotic height, and advanced tumor stage significantly increase the probability of following a severe and persistent LARS trajectory. Recognizing these factors as probabilistic risks rather than absolute determinants may facilitate early tailored interventions and enhance personalized postoperative recovery.

## 1 Introduction

Rectal cancer is one of the most common malignancies of the digestive system. With advances in surgical techniques, sphincter-preserving procedures, particularly low anterior resection, have become increasingly prevalent (1). While these approaches improve oncologic outcomes and help preserve anal function, they are often accompanied by postoperative functional impairments collectively known as low anterior resection syndrome (LARS), which is characterized by symptoms such as fecal incontinence, urgency, and increased bowel frequency (2, 3). The widespread use of low and ultralow anterior resection, in combination with neoadjuvant chemoradiotherapy, has led to a growing number of patients at risk of developing LARS (4). Management strategies for LARS include pharmacological therapy, biofeedback training, and sacral nerve stimulation (5). However, these interventions are underutilized in clinical practice, with only a small proportion of patients receiving them (6). From a surgical standpoint, the incidence of preventive stoma creation has risen, largely due to the increased use of low anterior resection and preoperative chemoradiotherapy. These temporary stomas are primarily employed to reduce the risk of anastomotic leakage following rectal cancer surgery (7).

Although numerous cross-sectional studies have investigated the prevalence and severity of LARS, longitudinal data remain limited. Increasing evidence suggests that the trajectory of LARS symptoms varies considerably among patients (8, 9). Some recover gradually over time, while others experience fluctuating or persistent symptoms (10). These heterogeneous patterns indicate that single-time-point evaluations may fail to capture the complexity and progression of postoperative bowel dysfunction.

Group-based trajectory modeling (GBTM) has emerged as a robust statistical approach for identifying distinct symptom evolution patterns within heterogeneous populations (11). By modeling symptom trajectories over time, GBTM can classify patients into latent subgroups based on their longitudinal symptom profiles. This method has been widely applied in chronic disease research, including oncology, mental health, and pain management, to reveal clinically relevant patterns and their predictors (12–15).

However, studies using GBTM to explore LARS progression are still scarce, particularly in specific subpopulations such as rectal cancer patients undergoing ileostomy and stoma reversal. The functional recovery patterns in this group may differ due to altered bowel physiology, delayed anastomotic function, and varied exposure to adjuvant treatments. Therefore, this study aimed to identify distinct LARS symptom trajectories in patients after ileostomy closure using GBTM, and to examine the associated clinical and treatment-related factors. Understanding these patterns may help clinicians better anticipate recovery and personalize postoperative care.

## 2 Materials and methods

### 2.1 Study population

A total of 134 patients diagnosed with rectal cancer and treated at the Fourth Hospital of Hebei Medical University between August 2022 and April 2024 were retrospectively enrolled in this retrospective, single-center study based on predefined eligibility criteria. Inclusion criteria were: (1) newly diagnosed rectal cancer; (2) treatment with low anterior resection involving presacral anastomosis and diverting ileostomy; (3) completion of ileostomy reversal; (4) informed consent provided by patients and their families; and (5) availability of complete clinical and follow-up data. Exclusion criteria included: (1) malignancies at other anatomical sites; (2) perianal conditions affecting anorectal function (abscesses or fissures); (3) severe psychiatric disorders; (4) recurrent or metastatic cancer; (5) permanent stoma; (6) underwent intersphincteric resection (ISR); and (7) inability to complete follow-up assessments. The study protocol was approved by the Ethics Committee of the Fourth Hospital of Hebei Medical University (Approval No. 2021111), and all procedures were conducted in accordance with the Declaration of Helsinki. Written informed consent was obtained from all participants prior to inclusion, ensuring adherence to established ethical standards.

### 2.2 Observation indicators

In this study, postoperative bowel dysfunction was assessed using the LARS score, which served as the primary outcome variable. Patients were categorized based on symptom severity: those with severe symptoms were assigned to the major LARS group, while individuals reporting no or only mild symptoms were placed in the no/minor LARS group. The LARS score was measured using a standardized and validated questionnaire specifically developed for evaluating bowel function following low anterior resection. Compared to the Wexner score, the LARS scale offers greater sensitivity and specificity in detecting anorectal dysfunction in patients who undergo low rectal surgery, and its reliability has been supported by multicenter validation studies across different countries (16). The instrument includes five items covering incontinence to flatus, incontinence to liquid stool, frequency of bowel movements, clustering of defecation, and urgency. The total score ranges from 0 to 42, with scores of 0–20 indicating no LARS, 21–29 indicating minor LARS, and 30–42 representing major LARS (17).

All curative rectal cancer surgeries were conducted in accordance with the principles of total mesorectal excision (TME), following established clinical guidelines (18). Diverting ileostomies were constructed as double-barrel terminal loop stomas in the right lower quadrant. All included patients underwent laparoscopic surgery using the double-stapling technique (DST) for anastomosis. The criteria for diverting ileostomy included preoperative chemoradiotherapy, a high risk of anastomotic leakage, or suspected compromise in anastomotic blood supply. Stoma care was managed by professional stoma nurses, who provided perioperative education, routine assessments, and postoperative maintenance. Reversal of the ileostomy was performed after clinical reassessment by the surgical team.

Demographic and clinical variables were recorded, including sex, age, postoperative TNM staging, use of neoadjuvant or adjuvant therapy, history of hypertension, heart disease, diabetes, body mass index (BMI), and the time interval to stoma reversal. For patients with locally advanced rectal cancer, neoadjuvant therapy was determined through multidisciplinary team (MDT) consultation and typically consisted of long-course chemoradiotherapy (28 fractions totaling 50.4 Gy or 30 fractions totaling 54 Gy) combined with 5-fluorouracil (5-FU)-based chemotherapy. Surgery was performed following completion of neoadjuvant therapy. For patients who declined radiotherapy but accepted chemotherapy, neoadjuvant chemotherapy alone was administered.

Tumor height was assessed preoperatively using sagittal T2-weighted magnetic resonance imaging (MRI). Anastomotic width was determined via contrast imaging of the lower gastrointestinal tract before ileostomy closure. All imaging was interpreted by experienced radiologists and reviewed by senior imaging specialists to ensure measurement accuracy. Anastomotic height was further evaluated by colonoscopy prior to stoma reversal.

### 2.3 Data collection

Low anterior resection syndrome scores were collected through structured telephone interviews or outpatient follow-ups. Stoma output was evaluated independently by specialized stoma care nurses. Prior to participation, informed consent was obtained from all patients. The LARS symptom questionnaire was administered to assess postoperative bowel function, and patients were screened in accordance with strict inclusion and exclusion criteria. To minimize potential bias, a dedicated follow-up team was established. All involved personnel received standardized training before the study commenced. Uniform questioning techniques were applied to reduce interviewer bias and enhance the reliability of patient-reported outcomes. General demographic data, surgical records, and imaging findings were extracted from the institutional electronic medical record system. Two independent investigators were responsible for organizing and verifying both clinical and follow-up data. Upon agreement, the verified data were entered into the study database.

### 2.4 Statistical methods

This study employed R software (R Project for Statistical Computing, Vienna, Austria; version 4.5.1) and Stata software (StataCorp, College Station, TX; version 18.0) for statistical analyses and figure generation. All continuous variables were tested for normality. Since none followed a normal distribution, they were presented as medians with interquartile ranges [M (IQR)], and comparisons between groups were conducted using the Mann–Whitney U test. Categorical variables were expressed as frequencies and percentages, and compared using the Chi-square test or Fisher’s exact test, as appropriate. GBTM was applied to LARS scores to identify distinct symptom trajectories (19). The optimal number of trajectories was determined by combining clinical interpretability with the Bayesian Information Criterion (BIC) as the model selection criterion (20), model entropy (>0.70), and a minimum subgroup size of 5%. Based on posterior probabilities, individuals with complete follow-up data were assigned to the most likely trajectory group. The polynomial order of the trajectories was determined using a step-down approach (resulting in a final order of 1, 2, 2) to best fit the data. Classification accuracy was assessed using the average posterior probability (AvePP) of group membership, with a value > 0.70 considered acceptable. Sensitivity analyses with randomized starting values were performed to confirm model stability. To investigate factors associated with different trajectory groups, univariable and multivariable multinomial logistic regression analyses were performed. It should be explicitly noted that the GBTM and regression analyses relied solely on baseline covariates; time-varying effects during the follow-up period were not modeled. A *P*-value < 0.05 was considered statistically significant.

## 3 Results

### 3.1 The symptom trajectories and baseline characteristics

**FIGURE 1:**
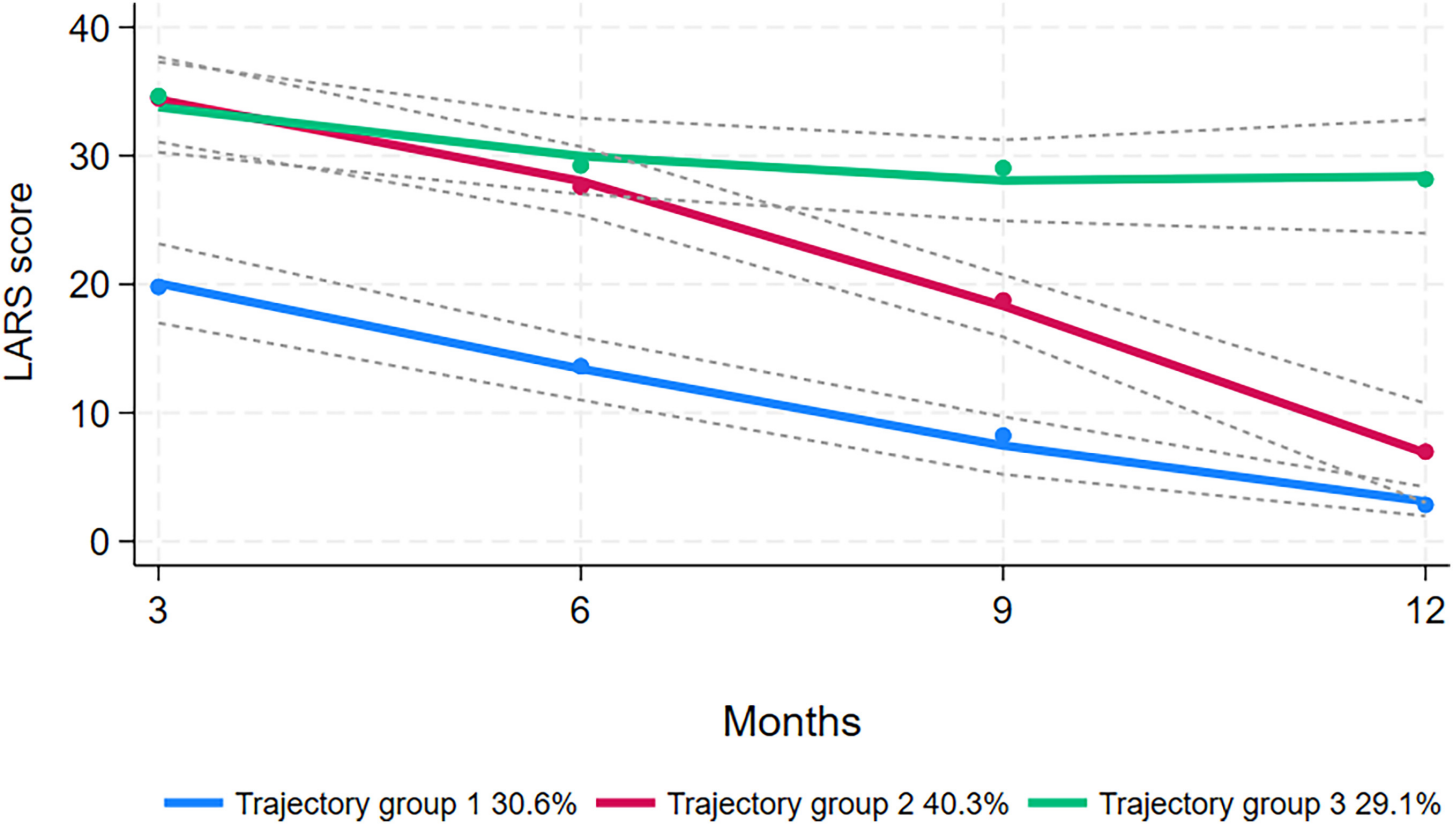
Low anterior resection syndrome (LARS) symptom trajectories over 12 months identified by group-based trajectory modeling. Solid lines represent the fitted trajectories; dashed lines indicate the associated 95% confidence intervals. LARS, low anterior resection syndrome.

This study included data from 134 Chinese patients. Using GBTM, three distinct symptom trajectories of LARS were identified: Group 1 (mild and recovering), Group 2 (moderate and fluctuating), and Group 3 (severe and persistent), as shown in Figure 1. Distribution across groups was as follows: 41 patients (30.6%) in Group 1, 54 (40.3%) in Group 2, and 39 (29.1%) in Group 3. The number of trajectory groups was determined based on clinical interpretability and the BIC. The BIC value for the final model was 1838.75. The polynomial order for the final model was set to 1, 2, 2 (linear for Group 1, and quadratic for Groups 2 and 3). The AvePP were 0.914, 0.893, and 0.921 for Groups 1, 2, and 3, respectively. Furthermore, the overall entropy of the model was 0.788. Significant differences were observed among the three trajectory groups in terms of T stage (*P* = 0.014), anastomotic height (*P* = 0.002), and receipt of preoperative radiotherapy (*P* < 0.001). However, no significant differences were found in age, sex, BMI, stoma closure interval, or comorbidities across the groups (Table 1).

**TABLE 1:** Baseline demographic and clinical characteristics across LARS trajectory groups.

Variables	Trajectory group 1	Trajectory group 2	Trajectory group 3	*P*-values
	*N* = 41	*N* = 54	*N* = 39	
Gender				0.986
Male	14 (34.15%)	19 (35.19%)	14 (35.90%)	
Female	27 (65.85%)	35 (64.81%)	25 (64.10%)	
Age, years	64.00 (57.00–71.00)	65.00 (57.00–70.00)	60.00 (54.50–70.50)	0.790*
Hypertension				0.954
Yes	21 (51.22%)	28 (51.85%)	19 (48.72%)	
No	20 (48.78%)	26 (48.15%)	20 (51.28%)	
Diabetes mellitus				0.236
Yes	3 (7.32%)	8 (14.81%)	8 (20.51%)	
No	38 (92.68%)	46 (85.19%)	31 (79.49%)	
Heart diseases				0.963
Yes	5 (12.20%)	6 (11.11%)	4 (10.26%)	
No	36 (87.80%)	48 (88.89%)	35 (89.74%)	
T stage				<0.001
T1–2	23 (56.10%)	11 (20.37%)	8 (20.51%)	
T3–4	18 (43.90%)	43 (79.63%)	31 (79.49%)	
N stage				0.265
N0	13 (31.71%)	8 (14.81%)	6 (15.38%)	
N1	4 (9.76%)	9 (16.67%)	6 (15.38%)	
N2	24 (58.54%)	37 (68.52%)	27 (69.23%)	
M stage				0.658
M0	38 (92.68%)	50 (92.59%)	38 (97.44%)	
M1	3 (7.32%)	4 (7.41%)	1 (2.56%)	
Preoperative radiotherapy				<0.001
Yes	3 (7.32%)	5 (9.26%)	14 (35.90%)	
No	38 (92.68%)	49 (90.74%)	25 (64.10%)	
Preoperative chemotherapy				0.170
Yes	8 (19.51%)	17 (31.48%)	15 (38.46%)	
No	33 (80.49%)	37 (68.52%)	24 (61.54%)	
Postoperative chemotherapy				0.100
Yes	28 (68.29%)	25 (46.30%)	21 (53.85%)	
No	13 (31.71%)	29 (53.70%)	18 (46.15%)	
Stoma closure intervals, days	233.00 (158.00–307.00)	201.50 (134.00–235.50)	189.00 (133.50–276.50)	0.181*
Anastomotic width, cm	1.70 (1.50–1.80)	1.78 (1.60–2.08)	1.76 (1.55–2.00)	0.372*
Tumor location, cm				0.110
≥5 cm	31 (75.61%)	37 (68.52%)	21 (53.85%)	
<5 cm	10 (24.39%)	17 (31.48%)	18 (46.15%)	
Anastomotic height, cm				0.003
≥4 cm	19 (46.34%)	9 (16.67%)	8 (20.51%)	
<4 cm	22 (53.66%)	45 (83.33%)	31 (79.49%)	
Body mass index, kg/m^2^				0.486
≥24.9	20 (48.78%)	33 (61.11%)	22 (56.41%)	
<24.9	21 (51.22%)	21 (38.89%)	17 (43.59%)	

Values are presented as *n* (%) for categorical variables and median (interquartile range) for continuous variables. The Kruskal–Wallis test was used to compare continuous variables among groups, and the Chi-square or Fisher’s exact test was used for categorical variables as appropriate. *The Mann–Whitney U test was employed for the purpose of comparing two groups. *P* < 0.05 was considered statistically significant. LARS, low anterior resection syndrome.

### 3.2 Longitudinal changes in LARS scores

After categorizing patients into no/minor LARS and major LARS groups, this study analyzed the distribution of LARS severity at 3, 6, 9, and 12 months postoperatively. At all time points, the differences in LARS categories across the symptom trajectory groups were statistically significant (*P* < 0.001), as shown in Table 2. Overall, LARS scores decreased over time, with lower scores observed at 12 months compared to 3 months post-surgery. In Group 1, most patients demonstrated progressive improvement, and the majority were classified as having no LARS by 12 months. Group 2 showed gradual symptom relief over time. In contrast, most patients in Group 3 continued to experience major LARS throughout the follow-up period, with 16 patients (41.03%) still categorized as having major LARS at 12 months.

**TABLE 2:** Distribution of LARS categories over time among symptom trajectory groups.

LARS categories	Trajectory group 1	Trajectory group 2	Trajectory group 3	*P*-values
	*N* = 41	*N* = 54	*N* = 39	
3 months				<0.001
No/minor LARS	37 (90.24%)	12 (22.22%)	6 (15.38%)	
Major LARS	4 (9.76%)	42 (77.78%)	33 (84.62%)	
6 months				<0.001
No/minor LARS	39 (95.12%)	30 (55.56%)	18 (46.15%)	
Major LARS	2 (4.88%)	24 (44.44%)	21 (53.85%)	
9 months				<0.001
No/minor LARS	41 (100.00%)	47 (87.04%)	20 (51.28%)	
Major LARS	0 (0.00%)	7 (12.96%)	19 (48.72%)	
12 months				<0.001
No/minor LARS	41 (100.00%)	54 (100.00%)	23 (58.97%)	
Major LARS	0 (0.00%)	0 (0.00%)	16 (41.03%)	

### 3.3 Multinomial logistic regression of influencing factors for LARS trajectories

Since Group 3 served as the reference, an OR < 1 represents a risk factor for severe symptoms, while an OR > 1 represents a protective effect. In the univariable analysis, T stage, anastomotic height, and receipt of preoperative radiotherapy were significantly associated with different LARS symptom trajectory groups. Preoperative radiotherapy showed significant differences in both comparisons between Group 1 vs. Group 3 and Group 2 vs. Group 3. Additionally, T stage and anastomotic height were significantly related to LARS trajectories when comparing Group 1 and Group 3. No significant associations were observed for age, sex, BMI, comorbidities (including hypertension, diabetes, and heart disease), stoma closure interval, N stage, M stage, or postoperative chemotherapy, as shown in Table 3.

**TABLE 3:** Univariable multinomial logistic regression for LARS trajectory groups (Reference: Group 3).

Variables	Trajectory group 1		Trajectory group 2	
	OR (95% CI)	*P*-values	OR (95% CI)	*P*-values
Gender				
Male	0.926 (0.369, 2.321)	0.87	0.969 (0.410, 2.291)	0.944
Female	Ref.		Ref.	
Age (per 1 year)	1.012 (0.970, 1.056)	0.57	1.004 (0.966, 1.044)	0.83
Hypertension				
Yes	1.105 (0.460, 2.657)	0.823	1.134 (0.497, 2.585)	0.766
No	Ref.		Ref.	
Diabetes mellitus				
Yes	0.306 (0.075, 1.252)	0.099	0.674 (0.229, 1.986)	0.474
No	Ref.		Ref.	
Heart diseases				
Yes	1.215 (0.301, 4.902)	0.784	1.094 (0.287, 4.169)	0.896
No	Ref.		Ref.	
T stage				
T1–2	Ref.		Ref.	
T3–4	0.202 (0.075, 0.545)	0.002	1.009 (0.363, 2.801)	0.987
N stage				
N0	Ref.		Ref.	
N1	2.437 (0.801, 7.416)	0.117	0.973 (0.302, 3.132)	0.963
N2	0.750 (0.189, 2.980)	0.683	1.095 (0.348, 3.443)	0.877
M stage				
M0	Ref.		Ref.	
M1	3.000 (0.299, 30.145)	0.351	3.040 (0.326, 28.315)	0.329
Preoperative radiotherapy				
Yes	0.141 (0.037, 0.541)	0.004	0.182 (0.059, 0.564)	0.003
No	Ref.		Ref.	
Preoperative chemotherapy				
Yes	0.388 (0.142, 1.061)	0.065	0.735 (0.310, 1.744)	0.485
No	Ref.		Ref.	
Postoperative chemotherapy				
Yes	1.846 (0.743, 4.589)	0.187	0.739 (0.323, 1.688)	0.473
No	Ref.		Ref.	
Stoma closure intervals (per 1 day)	1.003 (0.999, 1.008)	0.158	0.999 (0.995, 1.004)	0.814
Anastomotic width (per 1 cm)	0.768 (0.277, 2.126)	0.611	1.122 (0.435, 2.890)	0.812
Tumor location (per 1 cm)	1.113 (0.969, 1.278)	0.129	0.966 (0.841, 1.110)	0.625
Anastomotic height, cm				
≥4 cm	3.347 (1.243, 9.010)	0.017	0.775 (0.269, 2.229)	0.636
<4 cm	Ref.		Ref.	
Body mass index, kg/m2				
<24.9	1.359 (0.563, 3.278)	0.495	0.824 (0.357, 1.901)	0.649
≥24.9	Ref.		Ref.	

OR, odds ratio; CI, confidence interval; Ref., reference category. Multinomial logistic regression models were conducted separately for each variable to assess associations with LARS symptom trajectory groups, using Group 3 as the reference category. For continuous variables (e.g., age, stoma closure interval, tumor location), ORs represent the change per unit increase.

In the multivariable analysis, even after adjusting for the effect of preoperative radiotherapy, T stage remained an independently significant predictor associated with worse LARS trajectories (Group 1 vs. Group 3: OR = 0.253, 95% CI: 0.086–0.741, *P* = 0.012). Similarly, preoperative radiotherapy maintained its independent significance (OR = 0.198, 95% CI: 0.048–0.821, *P* = 0.026), alongside anastomotic height (OR = 3.929, 95% CI: 1.305–11.831, *P* = 0.015). For the comparison between Group 2 and Group 3, only preoperative radiotherapy was significantly associated (OR = 0.175, 95% CI: 0.055–0.551, *P* = 0.003), whereas T stage and anastomotic height were not statistically different between these two groups, as shown in Table 4.

**TABLE 4:** Multivariable multinomial logistic regression analysis of factors associated with LARS symptom trajectory groups (Reference: Group 3).

Variables	Trajectory group 1		Trajectory group 2	
	OR (95% CI)	*P*-values	OR (95% CI)	*P*-values
T stage				
T1–2	Ref.		Ref.	
T3–4	0.253 (0.086, 0.741)	0.012	1.353 (0.467, 3.920)	0.578
Preoperative radiotherapy				
Yes	0.198 (0.048, 0.821)	0.026	0.175 (0.055, 0.551)	0.003
No	Ref.		Ref.	
Anastomotic height, cm				
≥4 cm	3.929 (1.305, 11.831)	0.015	0.853 (0.280, 2.595)	0.779
<4 cm	Ref.		Ref.	

OR, odds ratio; CI, confidence interval; Ref., reference category. Multivariable multinomial logistic regression was conducted to examine factors independently associated with trajectory group 1 and group 2, using group 3 as the reference.

## 4 Discussion

This study identified three distinct symptom trajectories of LARS following ileostomy reversal in patients with rectal cancer: mild and recovering, moderate and fluctuating, and severe and persistent. Using GBTM, we observed that LARS symptoms exhibit heterogeneous evolution over time. Notably, a subset of patients continued to report severe symptoms even at 12 months postoperatively. Significant differences in T stage, anastomotic height, and preoperative radiotherapy were observed among the trajectory groups, suggesting a potential association between these clinical factors and functional recovery following low anterior resection.

Several studies have longitudinally examined symptom progression of LARS following rectal cancer surgery. Al-Rashid et al. employed GBTM to identify three major LARS trajectories over an 18-months postoperative period (21). They observed that patients initially presenting with moderate to severe LARS, but without neoadjuvant therapy, intersphincteric resection, or ileostomy, were more likely to demonstrate substantial functional improvement, whereas those with these risk factors tended to experience persistently high LARS scores. Consistent with these findings, our study also suggests that preoperative radiotherapy may be associated with less favorable LARS symptom trajectories. However, while Al-Rashid et al. primarily evaluated the impact of surgical techniques, our study focused more on anatomical and oncological parameters. Specifically, we found that a higher anastomotic height may predict improved functional recovery. Differences in results may partly reflect variations in patient selection and surgical consistency, as our cohort exclusively included patients who had undergone ileostomy reversal. In another study, He et al. conducted a long-term follow-up based on the FOWARC randomized controlled trial, with a median follow-up duration of 83 months (22). They observed partial recovery of anorectal function over time and identified preoperative radiotherapy as a predictor of poor functional outcomes. Importantly, 3.4% of patients eventually required secondary stoma creation due to persistent and severe dysfunction, underscoring the potential long-term burden of LARS and the need for proactive monitoring and intervention in high-risk individuals. Verkuijl et al. also reported a high prevalence of major LARS in patients after rectal cancer surgery (23). However, their multivariable analysis indicated that these outcomes were not directly attributable to the presence of a temporary colostomy or ileostomy. Instead, the clinical factors prompting diversion, such as lower anastomotic height, neoadjuvant radiotherapy, and anastomotic leakage, were the primary contributors to impaired bowel function. This aligns with our findings that preoperative radiotherapy and anastomotic height were significantly associated with LARS symptom trajectories, reinforcing the concept that LARS is a multifactorial condition rather than one driven solely by surgical technique. Moreover, Verkuijl et al. found that the timing of stoma reversal did not significantly affect long-term functional outcomes or quality of life, which is consistent with our observation that stoma closure interval did not differ significantly among trajectory groups. These findings suggest that individualized treatment planning should focus more on preoperative factors such as radiotherapy and anastomotic height rather than the type or timing of diversion procedures.

The heterogeneity of LARS symptom trajectories observed in this study is likely attributable to a multifactorial interaction among oncological characteristics, anatomical alterations, and treatment-related effects. Preoperative radiotherapy has been identified in multiple studies as a predictor of greater symptom severity and duration (24, 25). Notably, Dulskas et al. demonstrated that patients can experience sustained bowel dysfunction even years after receiving chemoradiotherapy, highlighting the profound and long-lasting detrimental effects of radiation on anorectal function (26). Whether administered before or after surgery, radiotherapy can induce pelvic tissue fibrosis, reduce rectal compliance, and damage the sacral plexus and autonomic neural pathways (27–29). These pathophysiological changes impair anorectal sensory and motor coordination and may underlie the persistence of symptoms in a subset of patients. Specifically, radiation-induced microvascular ischemia can lead to irreversible collagen deposition, severely restricting the distensibility of the neorectum (30). However, it is important to emphasize that radiotherapy should be interpreted as a risk stratifier rather than a deterministic factor. Although receiving radiotherapy significantly increases the probability of experiencing severe and prolonged LARS, it does not inevitably guarantee a poor functional outcome, as individual recovery patterns remain heterogeneous. Anastomotic height also represents a key mechanistic factor identified in this study. Lower anastomoses are associated with diminished neorectal reservoir function and may disrupt the rectoanal inhibitory reflex (31). In cases involving intersphincteric resection, direct impairment of the sphincteric apparatus may further elevate the risk of LARS (3). Additionally, T stage was significantly associated with LARS symptom trajectories. Locally advanced tumors, indicative of deeper invasion, often necessitate more extensive total mesorectal excision and pelvic dissection (32). These procedures increase the likelihood of incidental injury to surrounding nerves and musculature, potentially resulting in compromised anorectal function and delayed recovery (33). Moreover, higher T stage is commonly linked with neoadjuvant treatment, which may exacerbate symptom burden through additional therapeutic insult. The gradual symptom improvement observed in our “mild and recovering” and “moderate and fluctuating” groups highlights the mechanisms of delayed functional adaptation. Over months, the colonic neorectum undergoes morphological dilation to improve reservoir capacity, while compensatory hypertrophy of the anal sphincters and neuroplastic adaptation of the enteric nervous system gradually restore a degree of reflex coordination (34).

This study supports the concept that LARS is driven by multiple interacting factors, highlighting the limitations of standardized postoperative care and underscoring the need for personalized management strategies. Clinically, individualized approaches should begin with preoperative risk stratification to identify patients more likely to experience adverse symptom trajectories–particularly those undergoing low anastomoses, with high T stage tumors, or receiving neoadjuvant radiotherapy. For these patients, early and targeted intervention is essential. Management should include structured and longitudinal monitoring of bowel function to detect delayed or non-recovering patterns. Such monitoring may include the use of validated clinical assessment tools such as the low anterior resection syndrome score, in conjunction with anorectal manometry, rectal compliance testing, and scheduled functional follow-ups (35–37). Concurrently, patients should be offered early pelvic floor rehabilitation and biofeedback therapy to enhance anorectal coordination and restore functional integrity (38, 39). Integrating these measures into routine postoperative care may enable clinicians to better anticipate persistent dysfunction, tailor interventions to individual needs, and ultimately improve functional outcomes and patient satisfaction.

This study has several limitations. First, it was based on a single center cohort with a small sample size, which may limit the generalizability of the findings. Differences in surgical techniques, postoperative care, and patient compliance could also have influenced the results. Larger multicenter studies are needed to confirm these findings. Second, while GBTM helps describe symptom patterns, it does not consider time-varying factors or allow for causal interpretation. Future studies could use joint modeling or machine learning to improve prediction. Third, the follow-up period was limited to 12 months after stoma reversal. While this duration is adequate for capturing early recovery trajectories, it does not fully represent long-term LARS, which is commonly defined as ≥3–5 years postoperatively. Extended longitudinal follow-up is necessary to determine whether these probabilistic trajectories stabilize, improve, or deteriorate over the true long term. Fourth, due to the retrospective nature of this study, we lacked detailed data on several key surgical and postoperative variables known to influence LARS, including anastomotic leakage, postoperative pelvic sepsis, the specific type of anastomosis, quality of TME, nerve preservation, readmissions, and functional complications after stoma closure. Although we adjusted for major oncological and anatomical factors, the omission of these surgical parameters is a limitation. Finally, this study did not include objective measures like anorectal manometry or rectal compliance testing. Combining these with symptom scores could improve understanding of LARS mechanisms. In the future, clinical trials based on symptom trajectories are needed to test whether personalized care can reduce the burden of LARS. Mechanistic studies on nerve damage and fibrosis after radiotherapy may also help identify new treatment targets.

## 5 Conclusion

In rectal cancer patients who underwent low anterior resection with diverting ileostomy, we identified three distinct patterns of LARS symptom progression over the 12-months period following stoma reversal. Patients not receiving preoperative radiotherapy appeared more likely to follow recovering trajectories, even if they initially presented with severe symptoms. Conversely, these risk factors significantly increase the probability of a patient following a severe and persistent symptom trajectory. These insights may enhance preoperative counseling and support more personalized treatment planning and postoperative expectations for rectal cancer patients undergoing temporary diversion.

## Data Availability

The raw data supporting the conclusions of this article will be made available by the authors, without undue reservation.
